# Report of the Atlanta Board of Health, 1885

**Published:** 1886-04

**Authors:** 


### REPORT OF THE ATLANTA BOARD OF HEALTH FOR 1885.

Upon making a careful examination of the Seventh Annual Report of the Board of Health of the city of Atlanta, for the year 1885, we are impressed with the efficient performance of duty, not only by the Board, but so far as can be determined from the records, by the several officers and employes of the sanitary department.

That all has not been accomplished which is desirable for the attainment of a hygienic status in accordance with the progressive spirit manifested by our municipal authorities in other matters, results from circumstances which are duly noted in the report, and yet call for increased zeal on the part of this branch of our city organization to effect satisfactory results.

In regard to the inspection of the food supply, with its adulterations and deteriorations, the suggestions made should be accompanied by vigorous efforts to secure the enforcement of competent regulations.

It would seem that a thorough testing of the quality of water furnished by the "so-called artesian" well, and an accurate estimate of the quantity furnished by the constant pumping process, should have enabled the Board to speak of it in other terms than as simply an experiment; and it must strike every reader that a grave responsibility rests with the Board of Health to recommend some better mode of securing an adequate supply of pure water for drinking and other purposes if this is not to be relied on.

In the treatment of the sewer question, we appreciate fully the difficulties by which the Board is environed, in view of the existence of badly constructed underground drains in some parts of the city, which are entirely unfit for sewerage purposes, and it would be advisable, from a hygienic standpoint, whatever view may be taken of it esthetically, to fall back upon the open privy system until the plan for a general system of sewers, which is recommended by the Board, may be practicable. We concur in the judgment of the Board that an underground cesspool, such as reported, is a nuisance that demands prompt abatement, and there is reason to believe that many of the sewers throughout the city are sending forth pestiferous emanations. We learn from the report that there is not an ordinance in our Code prescribing the conditions for plumbing. "Reform radical and prompt is sorely needed here."

It strikes us as an inexcusable oversight that no attention has been given in this report to the hygienic conditions of our public school buildings, where so many children spend a large part of the day.

The total number of deaths from disease in this city for the year 1885 was 1,150, and of the 278 deaths in June and July, 158 or 56.83 per cent. were among children under five years of age, with a large preponderance of fatality among the colored population in this class, as well as in those over five years of age throughout the other months of the year.

